# Study of the Lithium Storage Mechanism of N‐Doped Carbon‐Modified Cu_2_S Electrodes for Lithium‐Ion Batteries

**DOI:** 10.1002/chem.202101818

**Published:** 2021-08-31

**Authors:** Guiying Tian, Chuanfeng Huang, Xianlin Luo, Zijian Zhao, Yong Peng, Yuqin Gao, Na Tang, Sonia Dsoke

**Affiliations:** ^1^ College of Chemical Engineering and Materials Science Tianjin University of Science and Technology 13th-Avenue 29, TEDA 300457 Tianjin P. R. China; ^2^ Institute for Applied Materials (IAM) Karlsruhe Institute of Technology (KIT) Hermann-von-Helmholtz-Platz 1 76344 Eggenstein-Leopoldshafen Germany; ^3^ Helmholtz-Institute Ulm for Electrochemical Energy Storage (HIU) Helmholtzstrasse 11 89081 Ulm Germany

**Keywords:** copper sulfide, synchrotron radiation diffraction, conversion mechanisms, capacitive storage, lithium-ion batteries

## Abstract

Owing to their high specific capacity and abundant reserve, Cu_*x*_S compounds are promising electrode materials for lithium‐ion batteries (LIBs). Carbon compositing could stabilize the Cu_*x*_S structure and repress capacity fading during the electrochemical cycling, but the corresponding Li^+^ storage mechanism and stabilization effect should be further clarified. In this study, nanoscale Cu_2_S was synthesized by CuS co‐precipitation and thermal reduction with polyelectrolytes. High‐temperature synchrotron radiation diffraction was used to monitor the thermal reduction process. During the first cycle, the conversion mechanism upon lithium storage in the Cu_2_S/carbon was elucidated by *operando* synchrotron radiation diffraction and in situ X‐ray absorption spectroscopy. The N‐doped carbon‐composited Cu_2_S (Cu_2_S/C) exhibits an initial discharge capacity of 425 mAh g^−1^ at 0.1 A g^−1^, with a higher, long‐term capacity of 523 mAh g^−1^ at 0.1 A g^−1^ after 200 cycles; in contrast, the bare CuS electrode exhibits 123 mAh g^−1^ after 200 cycles. Multiple‐scan cyclic voltammetry proves that extra Li^+^ storage can mainly be ascribed to the contribution of the capacitive storage.

## Graphical abstract

**Ad LIB**: In this study, thermal carbon reduction of CuS into Cu_2_S was monitored by high‐temperature synchrotron radiation diffraction (SRD). As LIB electrode material, Li^+^ storage intermediate Cu_1.8_S is confirmed by *operando* methods. The result shows that some original Cu_2_S remains unreacted after the first cycle, and carbon modification can improve the cycling stability of Cu_2_S for LIBs in the long‐term cycle.

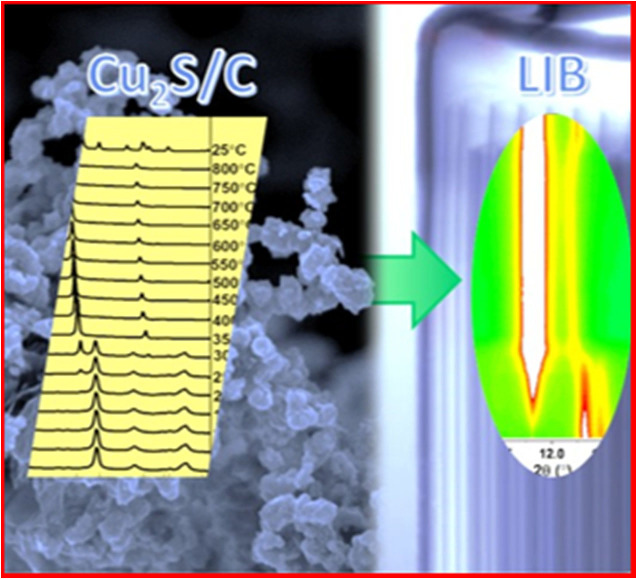

## Introduction

Nowadays, the rapid consumption growth of consumer electronics and electric vehicles requires a great amount of energy accumulator.^[1]^ To meet the request, tremendous efforts have been devoted to developing secondary batteries with high energy/power density, long lifespan and especially low cost.[^2^, ^3^] Owing to their outstanding electrochemical performance, flexible scale and no memory effect, LIBs dominate the current market. As a core component, the electrode materials determine the fundamental performance of LIBs. Achieving high specific capacity and long cycle life are the most important criteria for suitable electrode materials.

Conversion‐type materials gained much interest as negative electrodes for LIBs due to their high theoretical capacities and low cost. In the past, many conversion‐type materials such as transition metal oxides/sulfides/phosphides have been studied for LIBs. Among them, the metal‐sulfide bonds in transition metal sulfides (TMSs) can be easier broken/formed during electrochemical cycles. It is beneficial to achieve relatively rapid redox kinetics in TMS electrodes for lithium storage.^[4]^ Numerous TMS materials (e. g., FeS_2_, SnS_2_, NiS, MnS, CuS and ZnS) have been studied.[^5^, ^6^, ^7^, ^8^, ^9^, ^10^, ^11^, ^12^, ^13^] Among them, copper sulfide (Cu_*x*_S, *x*=1 or 2) has a wide variety of structures ranging from Cu‐poor to Cu‐rich phases, at least five stable phases at room temperature. As the electrode materials of LIBs, CuS and Cu_2_S exhibit theoretical capacities of 564 and 337 mAh g^−1^, respectively.^[14]^ Due to the smaller bandgap (1.21 eV), Cu_2_S has higher conductivity than that of CuS (2.42 eV).^[15]^ Park et al. reported that the Cu‐rich sulfides (Cu_*x*_S, *x*≥1.6) have a unit cell consisting of strong Cu−S bonds without S−S bonds. This structure is prone to external stress/strain that can result in bond cleavage as well as decomposition.^[16]^ Controlling microstructure of the electrode materials and designing porous morphology of building blocks can also relieve the volume fluctuation during the cycling process.[^17^, ^18^] Up to now, various structures of Cu_*x*_S (1D rods and tubes, 2D plates and sheets, and 3D spheres and flowers) have been fabricated as electrode materials.[^19^, ^20^] However, there are few reports using *operando* methods to clarify the initial insertion/phase evolution of the Cu_2_S during Li^+^ storage.

However, very few TMSs satisfy the criteria in regard to capacity performance and working life for commercial applications. The main problem is the redox potential hysteresis, as evidenced by the large potential difference between the cathodic and anodic half‐cycles. The irreversible capacity loss is due to the irreversible phase transformation and poor compatibility with electrolytes (dissolution of formed Li_2_S).[^21^, ^22^, ^23^, ^24^, ^25^, ^26^, ^27^] Obeying the similar electrochemical process of conversion electrodes, Cu_2_S inevitably suffers severe electrode pulverization and capacity fading during the cycling process, limiting its practical application.^[26]^ The polysulfides LiS_*x*_ (1<*x*<4) is easily dissolved into the organic electrolyte, resulting in poor capacity retention.^[28]^ In order to overcome these problems, three strategies have been proposed: 1) downsizing the Cu_2_S particles to the nanoscale, which can significantly reduce the volume change and mechanical stress during the conversion reaction; 2) applying a carbonaceous modification to the surface of Cu_2_S particles, which can avoid the direct contact of the material with the electrolyte and prevent the dissolution of LiS_*x*_;[^20^, ^22^] 3) loading heteroatom‐doped carbon, which is further beneficial for Li^+^ ions/electrons transport and wettability with liquid electrolytes;[^29^, ^30^, ^31^] 4) controlling the cycling conditions (by applying elevated temperatures, limiting the cycled potential range, pre‐cycling and pre‐lithiation of the electrode), which suppress the charge loss and stabilize conversion‐type electrodes.^[32]^ Based on these methods, an engineering strategy of Cu_2_S nanoparticles with heteroatom‐doped carbon is important to improve this material‘s cycling performance in LIBs.

In this study, we employed a simple co‐precipitation method to prepare nanosized CuS particles, which were then modified with N‐doped carbon derived from polyelectrolytes. A homogenous carbon coating could suppress the surface reactions between Cu_2_S and carbonate‐based electrolytes to retain its structural integrity and Cu_*x*_S displacement mechanism. The influence of N‐doped carbon loading on the electrochemical performance is evaluated by comparing the bare CuS and N‐dope Cu_2_S/C samples. Further, the conversion mechanism is analyzed using *operando* synchrotron radiation diffraction (SRD) and in situ X‐ray absorption spectroscopy (XAS) to unveil the phase evolution during Li^+^ storage. By combining the advantages of a short charge transport pathway and stable carbon loading, the as‐obtained Cu_2_S/C nanocomposite could serve as high‐performance anode materials for LIBs.

## Results and Discussion

### Synthesis and characterization of Cu_2_S/C

**Figure 1:**
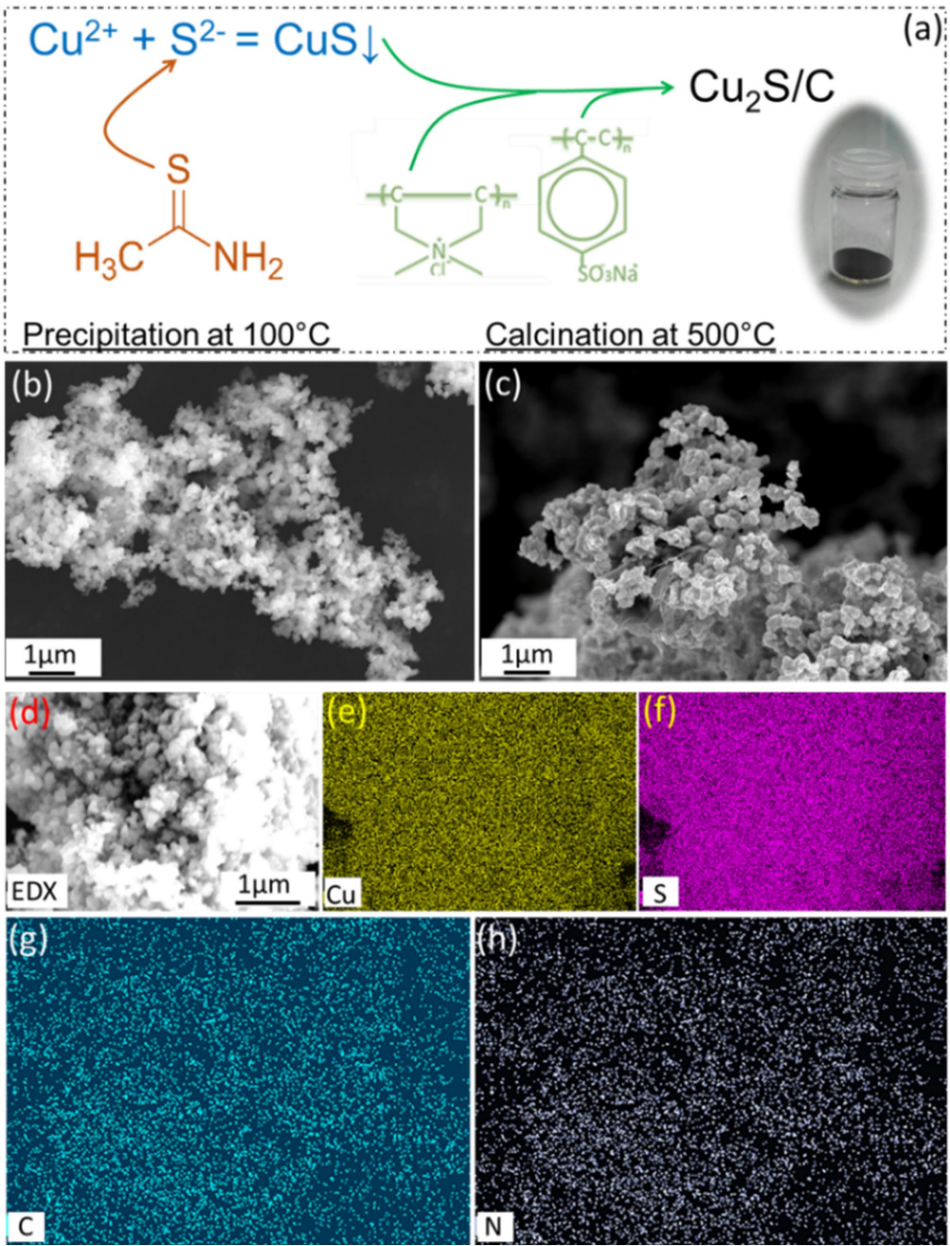
Schematic illustration of Cu_2_S/C preparation. SEM images of CuS and SEM image with corresponding EDX elemental mapping of the Cu_2_S/C (Cu/S/C/N).

In this study, the CuS precursor was prepared through a co‐precipitation method, as illustrated in Figure 1a. In boiling water, thioacetimidic acid provides a sulfur source (S^2−^) with dissolved Cu^2+^ to form insoluble CuS sediment in a weak alkaline environment. The Cu_2_S/C was achieved by carbonization and thermal reduction with CuS precursor and polyelectrolytes at 500 °C under an inert atmosphere (the details of the synthesis and characterization were given in the Supporting Information). The morphologies of the samples were characterized by SEM. Figure 1b displays the CuS cluster, which is stacked by a large number of primary CuS grains (average size of ∼90 nm). Figure 1c displays the Cu_2_S/C nanoparticles obtained after electrostatic assembly and thermal reduction, which retains the original size of the CuS. Such hierarchical architecture is beneficial for the infiltration of the electrolyte with fast charge transfer. Figure 1d–h displays the SEM image and corresponding EDX mappings of the Cu_2_S/C. These images clearly show that the Cu, S, C and N are distributed homogeneously over the Cu_2_S/C composite.

**Figure 2:**
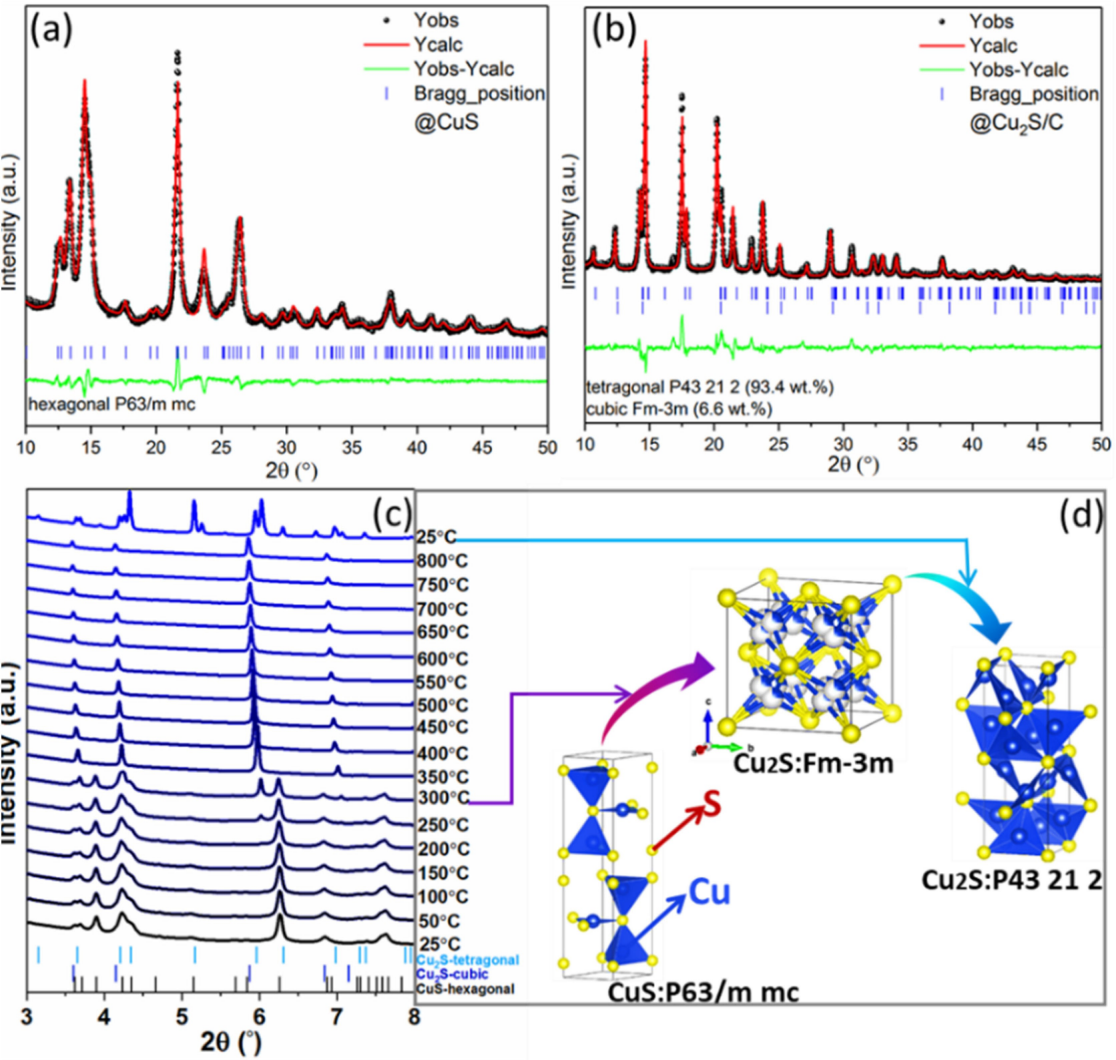
Rietveld refinement of the structural model based on the XRD patterns of a) CuS and b) Cu_2_S/C by using Mo_Kα1_ radiation (*λ*=0.70932 Å). c) HT‐SRD of the Cu_2_S/C precursor and d) the illustration of phase transition by using DESY synchrotron diffraction (*λ*=0.20737 Å).

CuS and Cu_2_S are considered as stable members of the Cu_*x*_S_*y*_ family. Despite their simple chemical formula, both CuS and Cu_2_S have complex crystal structures.^[33]^ In order to clarify the phase change during the carbonization process, a series of structural characterization was performed. A room‐temperature XRD pattern, as shown in Figure 2a, confirms that the CuS sample is a hexagonal phase with the space group *P*6_3_/*mmc* (registry no.: ICSD 41911, *a*=3.7938 Å, *c*=16.3410 Å). After carbonization at 500 °C, the mixing phases (tetragonal phase (registry no.: ICSD 95 398, *P*4_3_2_1_2, 93.4 wt %)+cubic phase (registry no.: ICSD 16 550, *Fm* $\bar{3}$ *m*, 6.6 wt %) of Cu_2_S were observed (Figure 2b). In order to understand the phase change during the thermal reduction, HT‐SRD was employed here. As shown in Figure 2c, diffraction peaks below 250 °C are corresponding to the hexagonal CuS phase. The reflections gradually shift to lower 2θ angles as the temperature rises, implying the simultaneous expansion of the unit cell. When the temperature rises to 350 °C, the reflections of CuS completely disappear, indicating the completion of the thermal reduction of CuS to Cu_2_S (Figure 2d). In addition, the reflections of cubic Cu_2_S continuously shift to lower angles with increasing temperature until 800 °C. After cooling down to room temperature, the cubic phase of Cu_2_S mainly transforms to its tetragonal phase, leading to formation of a biphasic Cu_2_S mixture.

**Figure 3:**
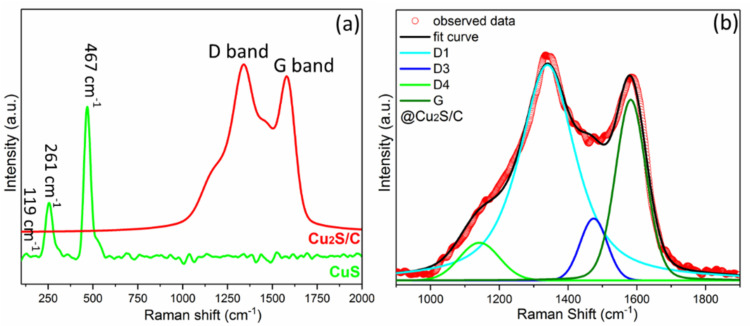
a) Raman spectra of CuS and Cu_2_S/C. b) Curve fit with band combination for the first‐order Raman spectrum of the Cu_2_S/C.

Herein, the coated N‐doped carbon in the Cu_2_S/C are not detected by XRD, due to the low amount and the amorphous nature. To uncover the properties of the loaded carbonaceous materials, Raman spectra were collected on the Cu_*x*_S samples in a wavelength of 100∼2000 cm^−1^. In Figure 3a, the band at 467 cm^−1^ is attributed to the S−S stretching mode (A_1g_ mode) of S^2−^ ions, and the narrow band also implies the high crystallinity.^[34]^ The bands at ∼261 and ∼119 cm^−1^ are also ascribed to the CuS.^[35]^ In contrast, predominant D (defective) and G (graphitic) bands are observed around 1315 cm^−1^ and 1580 cm^−1^ in the Cu_2_S/C spectrum, which suggests that Cu_2_S particles are fully coated with carbon material.[^36^, ^37^] In addition, the high ratio of G and D bands (*I*_G_/*I*_D_) for the Cu_2_S/C sample implies a high proportion of sp^2^‐conjugated carbon. In Figure 3b, the fitted *I*_G_/*I*_D_ value of ∼0.89 for the Cu_2_S/C is higher than the threshold value (0.52) for electron‐conductive carbon,[^38^, ^39^] indicating that the Cu_2_S/C nanoparticles are covered by the conductive carbon layer. Such carbon loading could serve as a buffer layer to avoid Li_*x*_S directly contacting electrolyte, further improving electrochemical cycling stability.^[40]^

XPS analysis was carried out on the Cu_2_S/C to uncover the surface chemistry of the pristine material. Figure S1a shows the XPS spectrum of Cu 2p. Peaks at 932.6 and 952.5 eV can be observed, while no satellite peak is found at the higher binding energy. Considering the Cu LMM‐2 peak at 917.1 eV, Cu^+^ is present in the Cu_2_S/C (Figure S1b).^[41]^ The spectrum of S 2p in Figure S1c can be deconvoluted to six peaks located at 162.1, 163.2, 163.8, 165.3, 168.3 and 169.6 eV, which are assigned to S^2−^ 2p_3/2_, S^2−^ 2p_1/2_, (S_2_)^2−^ 2p_3/2_, (S_2_)^2−^ 2p_1/2_, (SO_4_)^2−^ 2p_3/2_ and (SO_4_)^2−^ 2p_1/2_. The first four peaks indicate the existence of surface S^2−^, and the other peaks correspond to (SO_4_)^2−^ impurity. In Figure S1d, the evidence of C−N bond implies that the N atoms have been successfully doped in the carbon lattice on the Cu_2_S surface. As shown in Figure S1e, the N 1s XPS spectrum can be deconvoluted into two peaks located at 398.8 and 400.5 eV, ascribed to the pyridinic N and pyrrolic N, respectively. The N heteroatoms, derived from amine functional groups of poly(diallyldimethylammonium chloride) (PDDA), could enhance the electron conductivity and wettability with electrolyte. It is thus expected to improve the electrochemical kinetics performance of Li^+^ storage.[^31^, ^42^]

### Study of the electrochemical mechanism of Li^+^ storage

**Figure 4:**
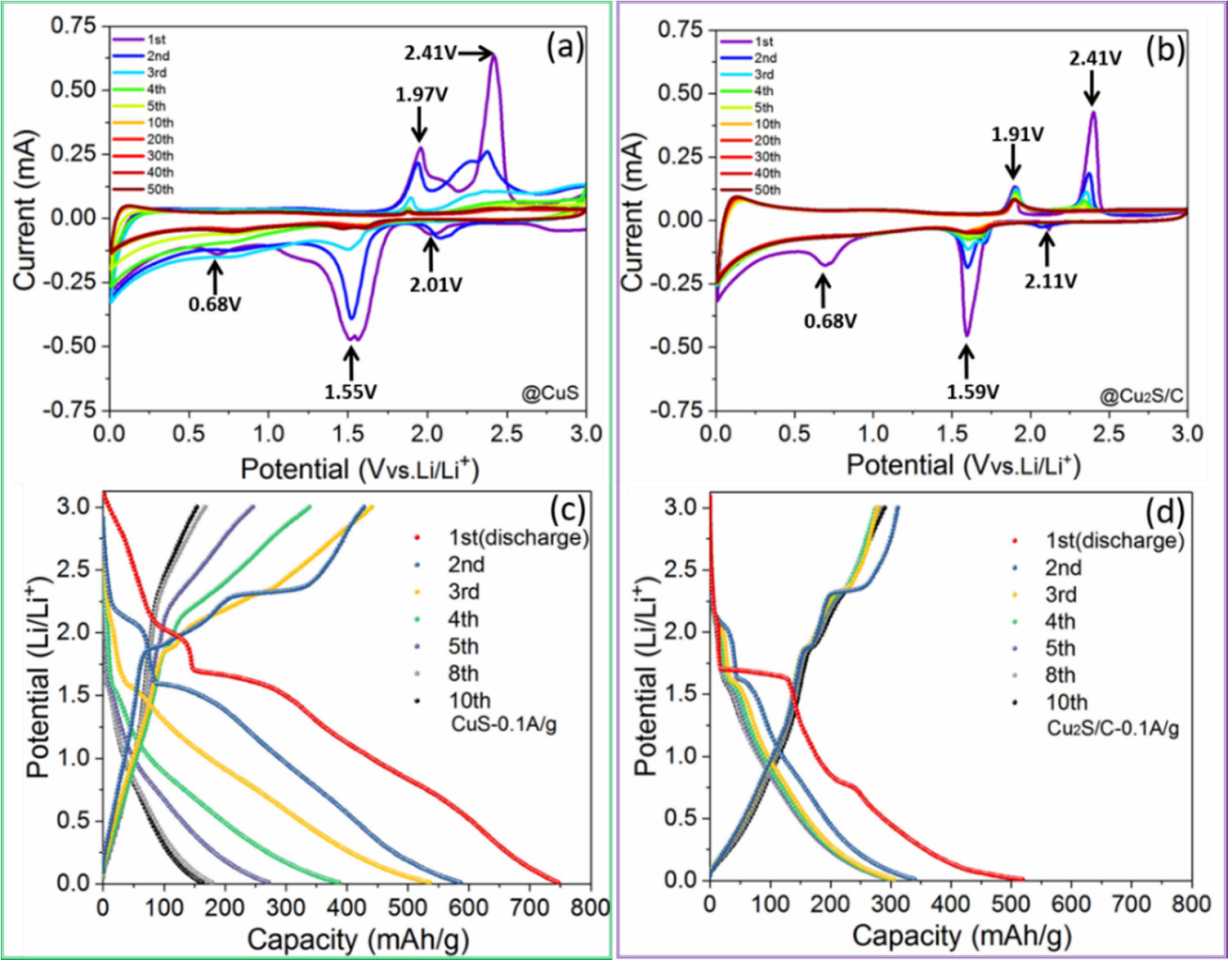
CV curves at a scan rate of 0.1 mV s^−1^ for a) CuS and b) Cu_2_S/C. GCD profiles at a current density of 0.1 A g^−1^ for c) CuS and d) Cu_2_S/C.

To further understand the electrochemical mechanism of Li^+^ insertion/desertion in carbon modified Cu_2_S, electrochemical characterization and mechanism analysis were conducted on the Cu_2_S/C composite and the bare CuS electrode (as a comparison). In this work, cyclic voltammetry (CV) and galvanostatic charge‐discharge (GCD) were recorded in a potential range of 0.01–3.00 V versus Li/Li^+^. As shown in Figure 4a, three cathodic peaks at 2.0, 1.5, and 0.7 V can be observed in the first CV curve of the bare CuS. In the 1st anodic profile, two peaks at 1.97 and 2.41 V are ascribed to the reversed conversion reaction to Cu_1.96_S.[^43^, ^44^] This indicates that the first cycle reaction is not completely reversible, and similar electrochemical behavior can be seen in the Cu_2_S/C electrodes. However, the Cu_2_S/C electrode shows an obvious Cu^+^/Cu^0^ conversion, evidenced by a sharp redox peak at 1.6 V. In the subsequent cycles, the characteristic current peaks are gradually vanishing, and Chung et al. ascribed this major apacity loss to Li_2_S formation and its dissolution.^[43]^ Further, the CV peaks of both CuS and Cu_2_S/C from the 5th to the 50th cycle gradually fade out, thus confirming the deeper pulverization and amorphous conversion of the materials. The Cu_2_S/C electrode shows a similar electrochemical behavior as CuS electrode (Figure 4b). By contrast, with the help of loading carbon, Cu_2_S/C electrode shows a relatively stable cycling performance.

In good agreement with the CV curves, the initial voltage plateaus at 1.6 V and 2.3 V in the GCD profiles gradually shorten and vanish after three cycles. Comparing the GCD profiles of the CuS and Cu_2_S/C at the specific current of 0.1 A g^−1^, one can see that the CuS electrode exhibits a relatively rapid capacity fading (Figure 4c). The specific capacity of CuS decreases from 746 mAh g^−1^ at 1st cycle to 172 mAh g^−1^ at 10th cycle. In contrast, the Cu_2_S/C displays a stable specific capacity of 311 mAh g^−1^ after 10th cycle, which is close to theoretical capacity (337 mAh g^−1^, Figure 4d). Of course, the additional capacity stored in the loaded carbon helps to increase the total capacity of the Cu_2_S/C.[^31^, ^45^] Thus, as a protective layer on the Cu_2_S particles, N‐doped carbon modification improves the structural‐ and electrochemical stability.

**Figure 5:**
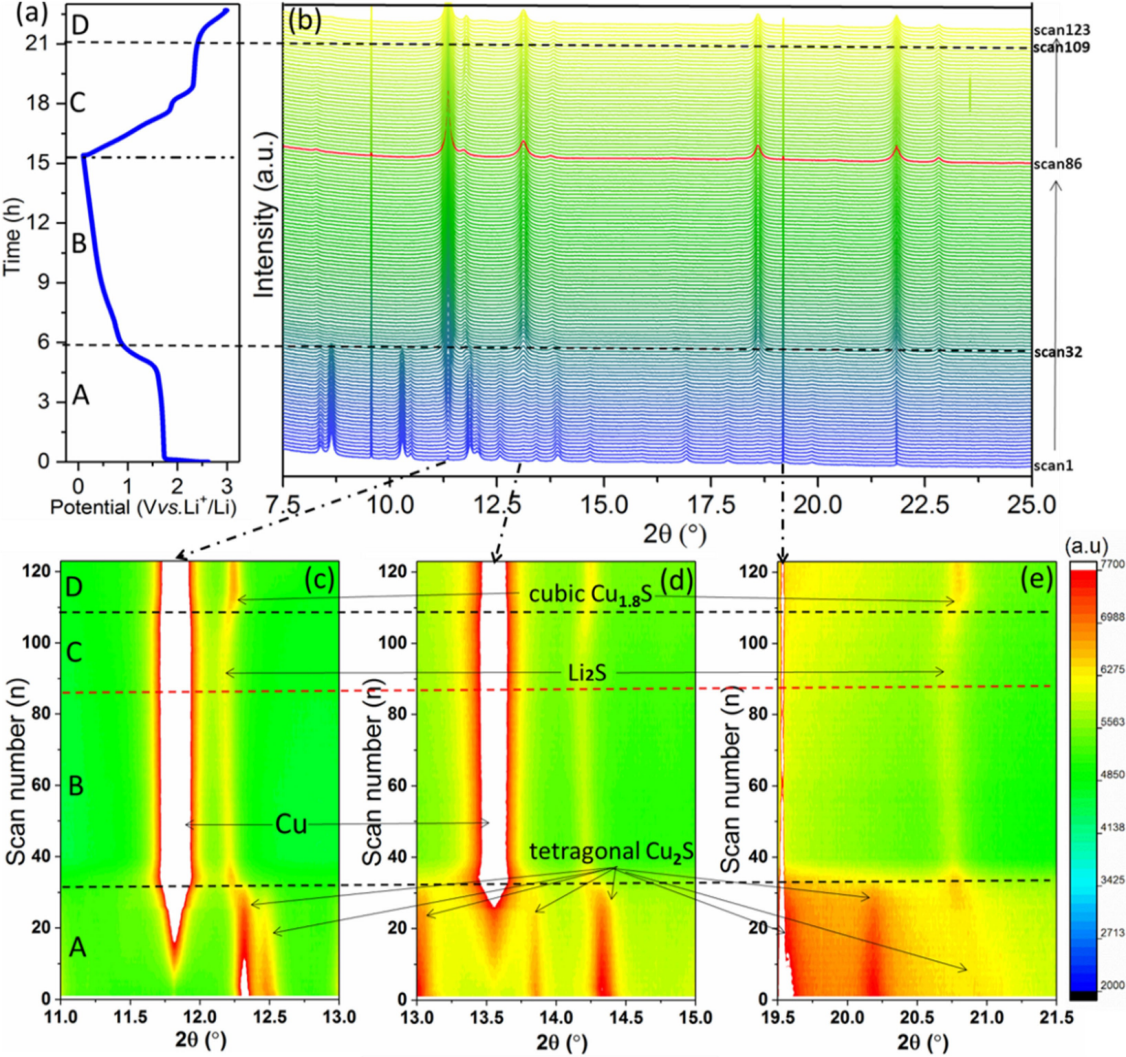
*Operando* SRD analysis of the Cu_2_S/C electrode in LIBs: a) GCD profile of the 1st lithiation at a current density of 50 mA g^−1^. b) *Operando* SRD patterns and c)–e) enlarged contour images of selected 2*θ* regions.

In 2002, Chung et al. reported the electrochemical study on CuS cathode as Li/CuS secondary cell at a voltage region of 1.5–2.6 V. Based on ex situ XRD/TEM analysis, three current peaks at the 1st cathodic scan in CV profiles (corresponding to potential plateaus at the 1st lithiation profile) are ascribed to the initial insertion (CuS+*x*Li^+^+*x*e^−^→Li_*x*_CuS), phase conversion (1.96Li_*x*_CuS+(2‐1.96*x*)Li^+^+(2‐1.96*x*)e^−^→Li_2_S+Cu_1.96_S; Cu_1.96_S+2Li^+^+2e^−^→Li_2_S+1.96Cu) and SEI formation, respectively.[^43^, ^44^] In the 1st anodic scan, two peaks at 1.97 and 2.41 V were ascribed to the reversed conversion reaction to Cu_1.96_S. In 2006, Tarascon's group applied in‐situ XRD/TEM to studying CuS electrode, and pointed out that a displacement reaction resulted in the growth and disappearance of copper dendrites of the initial CuS material. The poor capacity retention over the voltage range (0–2.5 V) is due to the partial solubility of formed Li_2_S into the electrolyte.^[46]^ Such previous works give some hints to study the (de)lithiation process of Cu_2_S/C in this work. The capacity loss of Cu_2_S during the initial cycles possibly arises from the irreversible conversion reaction and the SEI formation. However, up to now, specific discussion about the conversion reaction based on *operando* SRD analysis for Li^+^ storage in Cu_2_S/C has not been reported. With the aim to elucidate the energy storage mechanism of the Cu_2_S/C, *operando* SRD analysis combined with electrochemical tests was carried out. Figure 5a and b show the potential profile of the first GCD and the 123 SRD patterns, respectively, which were synchronously recorded. To clarify the multiple scans including phase conversion and lithiation, the first GCD is divided into four steps (steps A–D). Correspondingly, Figure S2 displays Rietveld analysis of selected scans relevant for the phase‐conversions at typical step. It is important to note that the initial reflections corresponding to metallic Cu and Li are due to the presence of the cupper current collector and lithium counter electrode in the testing cell.

In step A (OCV to 0.9 V, scan 1–32), the conversion occurs. The GCD profile exhibits a distinct potential plateau at ∼1.6 V, corresponding to the conversion process Cu^+^/Cu^0^, also evidenced by the first cathodic peak at 1.6 V in the CV tests (Figure 4b). At the end of step A, the reflections of the Cu_2_S phase show a rapid intensity decline until they completely vanish at scan32 (Figure S2a vs. b). At the same time, the intensity of the reflection corresponding to reduced metallic Cu increases suddenly (i. e., at 11.7° (Figure 5c) and 13.5° (Figure 5d)). In step B (0.9 to 0.01 V, scan 33–87, Figure S2b), the reflections of cubic Li_2_S and Cu remain stable. In the enlarged image of Figure S3, the positions of the reflection related to the cubic Li_2_S (Li_*x*_S) gradually shifts to lower 2*θ* angles, and the unit cell of Li_*x*_S enlarges with increasing *x* value due to the continuous lithium insertion process.[^22^, ^45^] In step C (0.01 to 2.3 V, scan 88–109, Figure S2c), the reflections of cubic Li_*x*_S go back to higher 2*θ*, indicating the reversibility of the conversion reaction: (2−*x*)Cu+Li_2_S↔Cu_2−*x*_S+2Li. However, the reflections of Cu still keep a high intensity, indicates a partial irreversibility of the reaction from Cu grains back to Cu_2−x_S (x→2).

Step D (2.3 to 3.0 V, scan 110–123) corresponds to the final conversion process. A new phase corresponding to cubic Cu_1.8_S (registry no.: ICSD 41142, space group of *Fm* $\bar{3}$ *m*, *a*=5.6843 Å) appears, indicating that the tetragonal Cu_2_S did not recover (Figure S2d). The copper current collector is not involved in the electrochemical reaction as its diffraction intensity is unchanged. Thus, it is noteworthy that the remained metallic Cu phase with high intensity indicates the existence of a large amount of residual copper grains. Such phase conversion reaction inevitably leads to structure destruction into nano‐sized grains. Meanwhile, the charge transfer resistance increases after the first cycle, as displayed in Figure S4. This could be ascribed to the generated Cu grains and Cu_1.8_S dispersed in the low‐conductive Li_2_S and around the polymeric SEI. This *operando* SRD study suggests that the first (de)lithiation cycle is an irreversible conversion process, thus responsible for the high initial irreversible capacity.

**Figure 6:**
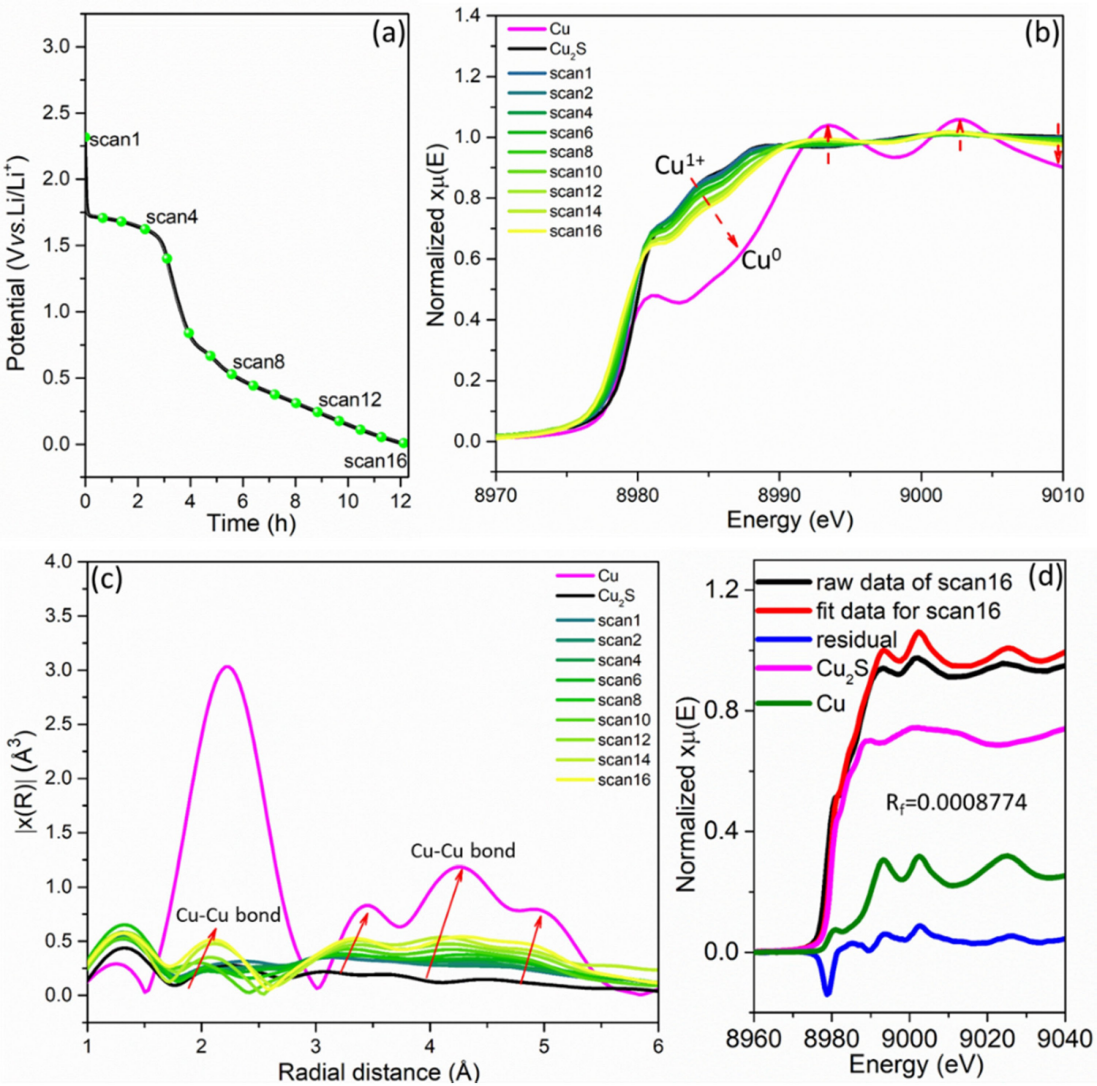
a) GCD profile during the 1st lithiation of the Cu_2_S/C electrode at a current density of 100 mA g^−1^. b) Corresponding normalized in‐situ XAS spectra at the Cu K‐edge. c) Corresponding Fourier transform of the recorded EXAFS‐spectra. d) Cu‐containing phase compositions at scan16 obtained by linear combination fitting.

In order to further understand the lithiation storage mechanism in the Cu_2_S/C electrode, in situ XAS spectra at the Cu K‐edge (8979 eV) have been collected during the 1st lithiation cycle performed at 100 mA g^−1^ from OCV to 0.01 V versus Li/Li^+^ (Figure 6a). Figure 6b shows that the Cu K‐edge of the pristine Cu_2_S/C electrode shifts to Cu^0^ energies (four red arrows) as the lithiation process goes on, confirming the conversion reaction Cu^1+^→Cu^0^. However, the mismatch of scan16 and the Cu‐foil reference suggests that the Cu_2_S does not completely convert into metallic Cu. Then, Cu K‐edge EXAFS Fourier transform curves are plotted in Figure 6c. As more Li^+^ is inserted, the potential decreases from OCV to 0.52 V (scan 1–8). Four distinct peaks of the Cu_2_S/C, corresponding to Cu−Cu 1st/2nd coordination shell, become more intensive. It corresponds to step A (OCV to 0.9 V, scan 1–32) in the *operando* SRD analysis, where Cu_2_S gradually converts as more Li^+^ ions are inserted. The estimation of the oxidation state of Cu in the Cu_2_S/C (according to reference spectra of Cu foil and Cu_2_S) is achieved through linear combination fitting of the Cu K‐edge XANES spectra (Figure 6d). The result proves that not all Cu_2_S/C material is involved in the 1st lithiation due to the fast‐cycling process, and 70.2 wt % of residual Cu_2_S still remains unreacted under this current density of 100 mA g^−1^.

**Figure 7:**
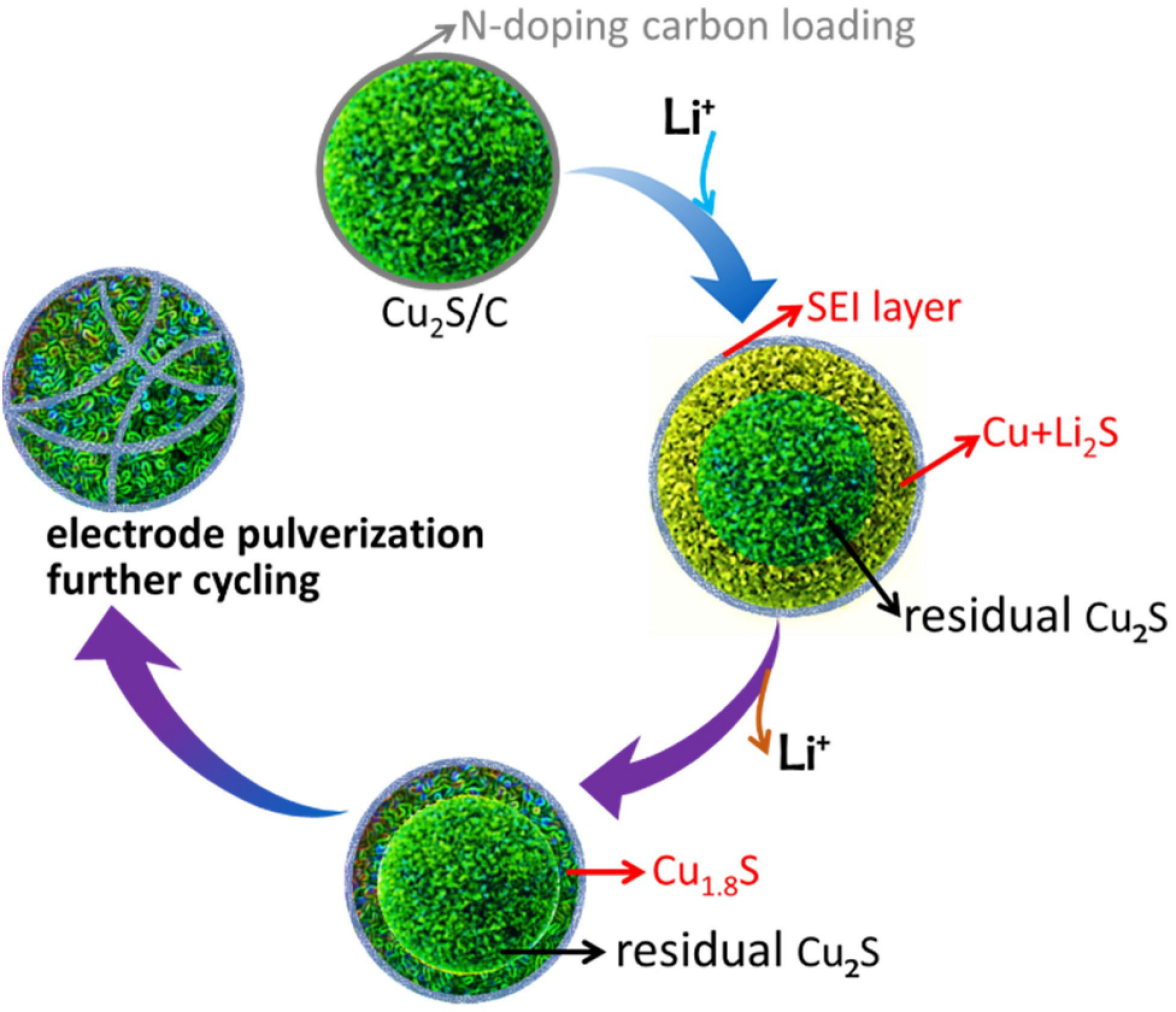
Schematic diagram of the Cu2S/C phase evolution during the 1st cycle.

Recently, Wang et al. reported that cycled Cu_2_S particles for LIBs turned polycrystalline grains after a fully cycled process by post‐mortem TEM analysis.^[47]^ According to the discussion above, one can summarize phase evolution process of Cu_2_S/C upon the first (de)lithiation process in the schematically shown in Figure 7. The corresponding electrochemical storage mechanism is proposed to be:

**Step A+B** (the first conversion reaction from OCV to 0.01 V vs. Li/Li^+^): *tetragonal* Cu_2_S+2Li^+^+2e^−^→Li_2_S+2Cu

**Step C+D** (the reverse conversion reaction from 0.01 to 3.0 V vs. Li/Li^+^): Li_2_S+1.8Cu→*cubic* Cu_1.8_S+1.8e^−^+2Li^+^

### Rate capability and long‐term cycling performance

**Figure 8:**
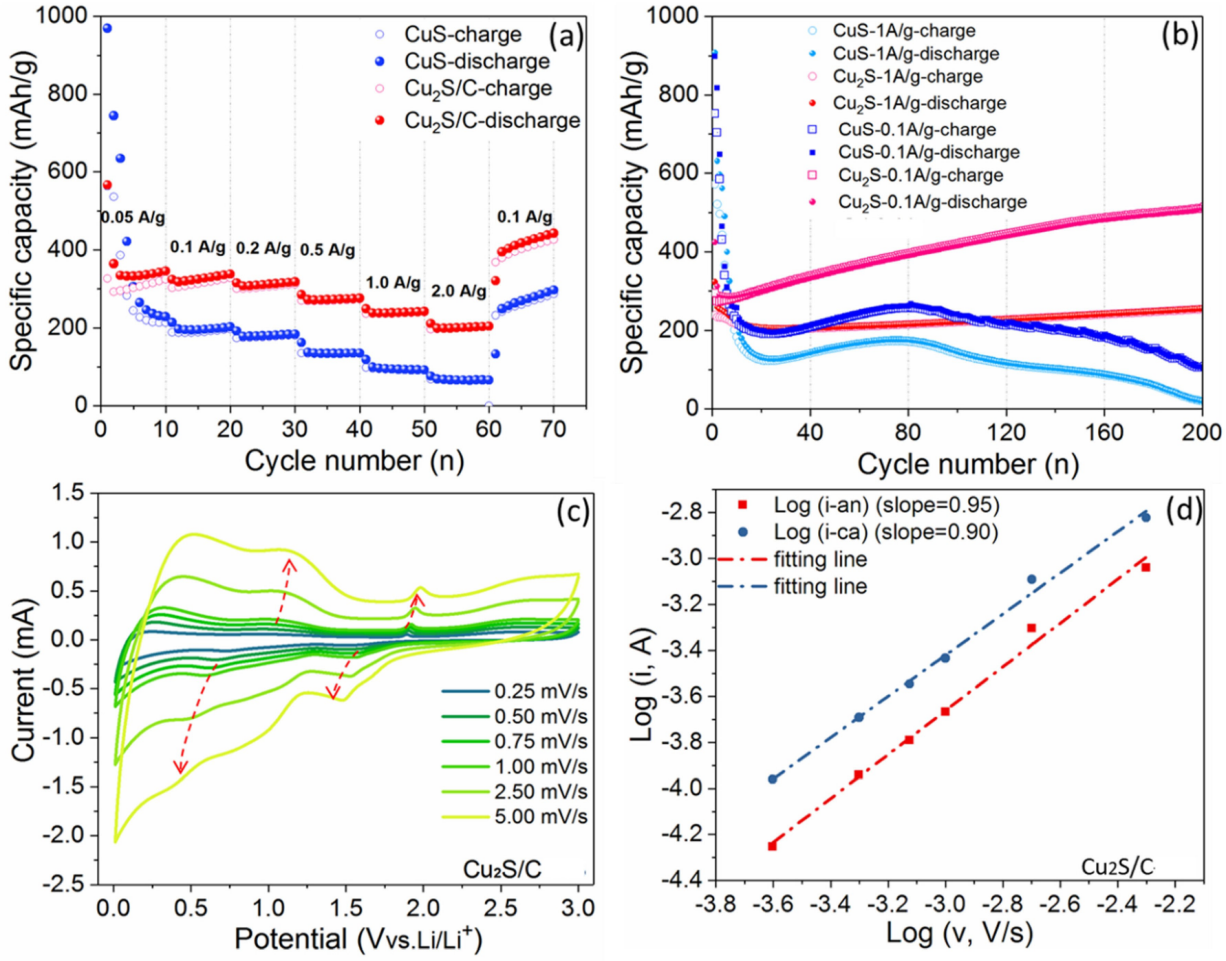
Electrochemical performance of CuS and Cu_2_S/C for lithium storage. a) Rate performance at different current densities. b) Cycling performance at current densities of 1.0 and 0.1 A g^−1^. c) CV curves at different scan rates from 0.25 to 5.00 mV s^−1^ for the Cu_2_S/C. d) Log(*i*) vs. log(*v*) plots and the fitted results based on CV peak currents for the Cu_2_S/C.

The performance of the electrodes has also been studied beyond the 1st cycle and in terms of rate capability. The CuS electrode demonstrates a fast capacity fading during the initial five cycles at the current density of 0.05 A g^−1^ (Figure 8a), suggesting an irreversible conversion of CuS electrode, and the SEI structure is not completely stabilized after the 1st cycle.^[15]^ The CuS electrode shows relatively lower capacities than those of Cu_2_S/C electrode at each current density (with specific capacities of 241, 200, 183, 135, 97, 68 and 351 mAh g^−1^ at current rates of 0.05, 0.1, 0.2, 0.5, 1.0, 2.0 and (back to) 0.1 A g^−1^, respectively). Although possessing high theoretical capacity (560 mAh g^−1^ of CuS vs. 337 mAh g^−1^ of Cu_2_S), the irreversible reaction blocks the achievement of the full potential capacity of the CuS electrode. In contrast, it is found that the Cu_2_S/C electrode exhibits high retention with discharge capacities of 342, 325, 315, 271, 243, 205 and 426 mAh g^−1^ at currents of 0.05, 0.1, 0.2, 0.5, 1.0, 2.0 and (back to) 0.1 A g^−1^, respectively. At very low current density, the actual discharge capacity of Cu_2_S/C electrode is larger than the theoretical capacity. In the conversion‐type electrode, besides the inherent conversion reaction, surface spin polarized capacitance of the reduced transition‐metal particles and reversible (de)formation of gelatinous polymer membrane catalyzed by transition‐metal contribute together to the extra capacity (beyond their theoretical capacity).[^48^, ^49^] Of course, the real detailed information beyond the theoretical capacity of Cu_2_S should be further clarified. The initial charge/discharge capacities of Cu_2_S/C are 566 and 328 mAhg^−1^, respectively, with an initial Coulombic efficiency of 57.9 %, which is higher than the pure CuS (52.4 %).

As shown in Figure 8b, long‐term cycling performance is evaluated at current densities of 1.0 A g^−1^ and 0.1 A g^−1^. The maximum capacity (262 mAh g^−1^ at 0.1 A g^−1^) of the CuS electrode can be obtained at around the 80th cycle. After 200 cycles, it displays the specific capacity of 123 mAh g^−1^. Differently, the Cu_2_S/C electrode delivers an initial discharge capacity of 425 mAh g^−1^ at 0.1 A g^−1^ and a steady increase in capacity within 200 cycles (523 mAh g^−1^ at 0.1 A g^−1^ at 200th cycle), which confirms that N‐doped carbon compositing effectively improves the cycling stability of copper‐sulfide material. This is also confirmed by previous studies about bare Cu_2_S electrode, and Table S1 summaries reported electrochemical performance of Cu_2_S electrode for LIBs. The rapid decrease in capacity after hundreds of cycles implies the necessity for carbon loading modification. As observed for other conversion‐type materials, the formed SEI layer among the pulverized electrode are beneficial for additional capacity. This is because that the reversible formation of a spin capacitor and the growth of a polymeric SEI film at low voltage could be sever as extra charge reservoirs.^[48]^ Notably, the increasing capacities could be a balance problem when configuring a full‐cell.

To evaluate the capacitive contribution to the lithium storage, multiple‐scan CV tests with varying scan rates from 0.25 to 5.0 mV s^−1^ were carried out. In Figure 8c, the redox peaks become broader as increasing the scan rates. Based on the power‐law relationship (*i_p_=av* ^*b*^, where *a* is a pre‐exponential index, and *b* reflects the contribution of different storage mechanisms).^[50]^ Herein, *b*=1 corresponds to a surface process, while *b*=0.5 is related to a diffusion‐controlled process. Figure 8d shows the *b* value determined by calculating the slope of log(*i*) versus log(*v*) between the CV peak current (*i_p_*) and scan rate (*v*), then one can distinguish the contribution of diffusive‐controlled process and surface‐controlled process to the total capacity. The result shows that *b* values are 0.95 and 0.90 for anodic scan and cathodic scan, respectively. This means that lithium storage is approximately a surface‐controlled (capacitive) behavior during the (de)lithiation processes.

## Conclusion

Carbon compositing modification is intensively employed to overcome the unstable phase transformation of conversion‐type electrodes. In this work, we have prepared N‐doped carbon modified Cu_2_S and applied Cu_2_S/C as an electrode material in rechargeable LIBs. The thermal reduction process of CuS to Cu_2_S, occurring at 350 °C, is evidenced by HT‐SRD. The Cu_2_S/C electrode shows a high specific capacity of 523 mAh g^−1^ at a current density of 0.1 A g^−1^ at the 200th cycle. The loading of N‐doped carbon can suppress Li_*x*_S dissolution and avoid excessive SEI formation. The excellent rate performance can be attributed to the nanoscale size of the active particles and to the conductive carbon modification on Cu_2_S particles. The capacitive‐controlled storage contributes to an additional capacity in the Cu_2_S/C. The lithium storage mechanism in the 1st cycle is discussed based on the *operando* SRD and in situ XAS analysis. We can conclude in general that operando techniques help to understand the lithium storage mechanism in Cu_2_S/C.

## Conflict of interest

The authors declare no conflict of interest.

## Supporting information

As a service to our authors and readers, this journal provides supporting information supplied by the authors. Such materials are peer reviewed and may be re‐organized for online delivery, but are not copy‐edited or typeset. Technical support issues arising from supporting information (other than missing files) should be addressed to the authors.

Supporting Information

## References

[1] J. G. Kim , B. Son , S. Mukherjee , N. Schuppert , A. Bates , O. Kwon , M. J. Choi , H. Y. Chung , S. Park , J. Power Sources 2015, 282, 299–322.

[2] B. Scrosati , J. Garche , J. Power Sources 2010, 195, 2419–2430.

[3] J. M. Tarascon , M. Armand , Nature 2001, 414, 359–367. 11713543 10.1038/35104644

[4] R. Kanno , M. Murayama , J. Electrochem. Soc. 2001, 148, A742.

[5] Y. Lin , Z. Qiu , D. Li , S. Ullah , Y. Hai , H. Xin , W. Liao , B. Yang , H. Fan , J. Xu , C. Zhu , Energy Storage Mater. 2018, 11, 67–74.

[6] M. R. Gao , Y. F. Xu , J. Jiang , S. H. Yu , Chem. Soc. Rev. 2013, 42, 2986–3017. 23296312 10.1039/c2cs35310e

[7] Q. Zhang , Z. Xu , B. Lu , Energy Storage Mater. 2016, 4, 84–91.

[8] J. Cui , S. Yao , J. K. Kim , Energy Storage Mater. 2017, 7, 64–114.

[9] Y. Xiao , J. Y. Hwang , I. Belharouak , Y. K. Sun , Nano Energy 2017, 32, 320–328.

[10] Y. Lin , Z. Qiu , D. Li , S. Ullah , Y. Hai , H. Xin , W. Liao , B. Yang , H. Fan , J. Xu , C. Zhu , Energy Storage Mater. 2018, 11, 67–74.

[11] D. Bresser , S. Passerini , B. Scrosati , Energy Environ. Sci. 2016, 9, 3348–3367.

[12] X. Wang , K. Chen , G. Wang , X. Liu , H. Wang , ACS Nano 2017, 11, 11602–11616. 29049876 10.1021/acsnano.7b06625

[13] P. Mei , M. Pramanik , J. Lee , Y. Ide , Z. A. Alothman , J. H. Kim , Y. Yamauchi , Chem. Eur. J. 2017, 23, 4344–4352. 28267228 10.1002/chem.201604159

[14] S. Foley , H. Geaney , G. Bree , K. Stokes , S. Connolly , M. J. Zaworotko , K. M. Ryan , Adv. Funct. Mater. 2018, 28, 1–8.

[15] Y. Xiao , D. Su , X. Wang , S. Wu , L. Zhou , Y. Shi , S. Fang , H.-M. Cheng , F. Li , Adv. Energy Mater. 2018, 8, 1800930.

[16] H. Park , J. Kwon , H. Choi , D. Shin , T. Song , X. W. D. Lou , ACS Nano 2018, 12, 2827–2837. 29505231 10.1021/acsnano.8b00118

[17] S. Yun , S. Bak , S. Kim , J. S. Yeon , M. G. Kim , X. Yang , P. V. Braun , H. S. Park , Adv. Energy Mater. 2019, 9, 1802816.

[18] P. Bhattacharya , M. Kota , D. H. Suh , K. C. Roh , H. S. Park , Adv. Energy Mater. 2017, 7, 2017.

[19] C. An , Y. Ni , Z. Wang , X. Li , X. Liu , Inorg. Chem. Front. 2018, 5, 1045–1052.

[20] J. Li , D. Yan , T. Lu , W. Qin , Y. Yao , L. Pan , ACS Appl. Mater. Interfaces 2017, 9, 2309–2316. 28032984 10.1021/acsami.6b12529

[21] H. H. Fan , H. H. Li , K. C. Huang , C. Y. Fan , X. Y. Zhang , X. L. Wu , J. P. Zhang , ACS Appl. Mater. Interfaces 2017, 9, 10708–10716. 28263060 10.1021/acsami.7b00578

[22] Y. Ma , Y. Ma , D. Bresser , Y. Ji , D. Geiger , U. Kaiser , C. Streb , A. Varzi , S. Passerini , ACS Nano 2018, 12, 7220–7231. 29940098 10.1021/acsnano.8b03188

[23] L. Wang , J. Wang , F. Guo , L. Ma , Y. Ren , T. Wu , P. Zuo , G. Yin , J. Wang , Nano Energy 2018, 43, 184–191.

[24] R. Zhang , Y. Wang , M. Jia , J. Xu , E. Pan , Appl. Surf. Sci. 2018, 437, 375–383.

[25] S. Gao , G. Chen , Y. Dall'Agnese , Y. Wei , Z. Gao , Y. Gao , Chem. Eur. J. 2018, 24, 13535–13539. 29904945 10.1002/chem.201801979

[26] Q. Wang , R. Zou , W. Xia , J. Ma , B. Qiu , A. Mahmood , R. Zhao , Y. Yang , D. Xia , Q. Xu , Small 2015, 11, 2511–2517. 25688868 10.1002/smll.201403579

[27] B. Qu , C. Ma , G. Ji , C. Xu , J. Xu , Y. S. Meng , T. Wang , J. Y. Lee , Adv. Mater. 2014, 26, 3854–3859. 24677348 10.1002/adma.201306314

[28] B. Jache , B. Mogwitz , F. Klein , P. Adelhelm , J. Power Sources 2014, 247, 703–711.

[29] Q. Chen , M. Ren , H. Xu , W. Liu , J. Hei , L. Su , L. Wang , ChemElectroChem 2018, 5, 2135–2141.

[30] D. Xu , C. Chen , J. Xie , B. Zhang , L. Miao , J. Cai , Y. Huang , L. Zhang , Adv. Energy Mater. 2016, 6, 1–7.

[31] Y. Ma , Y. Ma , D. Geiger , U. Kaiser , H. Zhang , G. T. Kim , T. Diemant , R. J. Behm , A. Varzi , S. Passerini , Nano Energy 2017, 42, 341–352.

[32] J. Asenbauer , A. Varzi , S. Passerini , D. Bresser , J. Power Sources 2020, 473, 228583.

[33] P. Kumar , R. Nagarajan , R. Sarangi , J. Mater. Chem. C 2013, 1, 2448. 10.1039/C3TC00639E PMC3683299 23781327

[34] M. Ishii , K. Shibata , H. Nozaki , J. Solid State Chem. 1993, 105, 504–511.

[35] T. Hurma , S. Kose , Optik 2016, 127, 6000–6006.

[36] S. Bose , T. Kuila , M. E. Uddin , N. H. Kim , A. K. T. Lau , J. H. Lee , Polymer 2010, 51, 5921–5928.

[37] J. Bai , X. Jiang , Anal. Chem. 2013, 85, 8095–8101. 23826825 10.1021/ac400659u

[38] A. Sadezky , H. Muckenhuber , H. Grothe , R. Niessner , U. Pöschl , Carbon 2005, 43, 1731–1742.

[39] F. Tuinstra , J. L. Koenig , J. Compos. Mater. 1970, 4, 492–499.

[40] G. Tian , Z. Zhao , A. Sarapulova , C. Das , L. Zhu , S. Liu , A. Missiul , E. Welter , J. Maibach , S. Dsoke , J. Mater. Chem. A 2019, 7, 15640–15653.

[41] H. Li , K. Wang , S. Cheng , K. Jiang , ACS Appl. Mater. Interfaces 2018, 10, 8016–8025. 29425016 10.1021/acsami.7b19138

[42] Y. Mao , H. Duan , B. Xu , L. Zhang , Y. Hu , C. Zhao , Z. Wang , L. Chen , Y. Yang , Energy Environ. Sci. 2012, 5, 7950–7955.

[43] J.-S. Chung , H.-J. Sohn , J. Power Sources 2002, 108, 226–231.

[44] X. Ding , S. Lei , C. Du , Z. Xie , J. Li , X. Huang , Adv. Mater. Interfaces 2019, 6, 1900038.

[45] G. Tian , Z. Zhao , A. Sarapulova , C. Das , L. Zhu , S. Liu , A. Missiul , E. Welter , J. Maibach , S. Dsoke , J. Mater. Chem. A 2019, 7, 15640–15653.

[46] A. Débart , L. Dupont , R. Patrice , J.-M. Tarascon , Solid State Sci. 2006, 8, 640–651.

[47] Y. Wang , X. Feng , Y. Xiong , S. Stoupin , R. Huang , M. Zhao , M. Xu , P. Zhang , J. Zhao , H. D. Abruña , ACS Appl. Mater. Interfaces 2020, 12, 17396–17405. 32208634 10.1021/acsami.9b21982

[48] H. Li , Z. Hu , Q. Xia , H. Zhang , Z. Li , H. Wang , X. Li , F. Zuo , F. Zhang , X. Wang , W. Ye , Q. Li , Y. Long , Q. Li , S. Yan , X. Liu , X. Zhang , G. Yu , G. Miao , Adv. Mater. 2021, 33, 2006629. 10.1002/adma.202006629 33576103

[49] Q. Li , H. Li , Q. Xia , Z. Hu , Y. Zhu , S. Yan , C. Ge , Q. Zhang , X. Wang , X. Shang , S. Fan , Y. Long , L. Gu , G. Miao , G. Yu , J. S. Moodera , Nat. Mater. 2021, 20, 76–83. 32807921 10.1038/s41563-020-0756-y

[50] G. A. Muller , J. B. Cook , H. S. Kim , S. H. Tolbert , B. Dunn , Nano Lett. 2015, 15, 1911–1917. 25654445 10.1021/nl504764m

