# Supplementary material for: Study of the Lithium Storage Mechanism of N‐Doped Carbon‐Modified Cu2S Electrodes for Lithium‐Ion Batteries
Source: Chemistry. 2021 Aug 31;27(55):13774–82. doi: 10.1002/chem.202101818 (PMC9400886; doi:10.1002/chem.202101818)
Supplement: Supplementary file 1 — Supporting Information [file CHEM-27-13774-s001.pdf]

Chemistry–A European Journal

Supporting Information

Study of the Lithium Storage Mechanism of N-Doped Carbon-Modified Cu_2S Electrodes for Lithium-Ion Batteries

Guiying Tian, Chuanfeng Huang, Xianlin Luo, Zijian Zhao,* Yong Peng, Yuqin Gao, Na Tang, and Sonia Dsoke*

Supporting Information

Experimental Section

Synthesis of CuS/C via co-precipitation

CuS nanoparticles were synthesized via a co-precipitation method. $Cu(NO_3)_2{\cdot}3H_2O$ (≥98.0%, Sigma-Aldrich) and thioacetimidic acid (C_2H_5NS, ≥99.0%, Sigma-Aldrich) were used in Cu:S molar ratio of 1.0:1.1. $Cu(NO_3)_2$ was firstly dissolved in deionized water. Afterwards, 10 wt.% thioacetimidic acid aqueous solution was dropwise added into the boiling 0.5M $Cu(NO_3)_2$ solution with vigorous stirring. The sediment, formed via an electrostatic assembly, was washed and centrifuged several times with deionized water and ethanol. After drying at 80 °C for 12 h, a black CuS precursor was obtained. A part of CuS precursor was calcinated at 500 °C for 3 h under argon flow, denoted as CuS. The rest of the CuS precursor was modified via the carbon loading method (see next session 2.2).

Synthesis of Cu_2S/C via thermal reduction

Based on the previous report,[1] 0.50 g CuS precursor was dispersed in 100 mL deionized water to form a homogeneous suspension. Then, 1.25 g poly(diallyldimethylammonium chloride) (PDDA, 20 wt.%, M_w<500000 Da, Aldrich) and 0.84 g poly(sodium-4-styrenesulfonate) (PSS, 30 wt.%, M_w<70000 Da, Aldrich) were respectively dispersed in the suspension under vigorous stirring. The obtained sediment was washed and carbonized at 500 °C for 3 h under argon flow, denoted as Cu_2S/C.

General materials characterization

Raman test was performed by a Raman spectrometer (LabRam Evolution HR, HORIBA Jobin Yvon) using 633 nm laser. XRD test was employed using an STOE STADI P X-ray powder diffractometer equipped with a Mythen1K detector and a Mo $K_{\alpha1}$ radiation (λ=0.70932 Å) in a 2θ range of 10°~50° in Debye–Scherrer geometry. Rietveld refinement was performed to analyze the diffraction data using FullProf software [2]. A field-emission scanning electron microscope (SEM, Merlin Carl Zeiss) was used to characterize the sample morphology. Energy-dispersive X-ray spectroscopy (EDX, Bruker, Quantax 400 SDD) was used to determine the elemental composition. X-ray photoelectron spectroscopy (XPS) was performed on a K-Alpha XPS instrument (Thermo-Fisher Scientific, East Grinstead). The K-Alpha charge compensation system was used to prevent any local charge buildup. All samples were analyzed using a monochromated Al K_α X-ray source (400 µm spot size). The spectra are referenced in binding energy to the C 1s peak (C–C, C–H) at 285 eV binding energy.

Operando test using synchrotron radiation diffraction

To monitor the phase evolution of the Cu_2S/C precursor during the thermal reduction process, *operando* high-temperature synchrotron radiation powder diffraction (HT-SRD) was employed at P02.1, DESY PETRA-III, Germany, using monochromatic synchrotron diffraction (λ = 0.20737(4) Å). Here, NaCl and LaB_6 were used as standard samples for the calibration of temperature and wavelength. The Cu_2S/C precursor was sealed in a quartz capillary under argon atmosphere and heated with a heating rate of 20 °C min^{-1} up to 800 °C. The as-obtained 2D image data were transformed to intensity-2θ data using Fit2D software.[2] Also, *in situ* X-ray absorption spectroscopy (XAS) spectra were collected in transmission geometry with the step-scan mode at the Cu K-edge (8979 eV). The Si(111) double-crystal monochromator equipped with a ionization chamber was optimized for the Cu edge absorption. Here, coin cells with glass windows (Li||LP30||Cu_2S/C) were assembled. The working electrodes were prepared by mixing Cu_2S/C (70 wt.%), carbon black (20 wt.%, Super P Li, Timcal Ltd.) and polytetrafluoroethylene (10 wt.%, PTFE beads, Aldrich), and then loaded on the steel mesh disk (ϕ 12 mm) as current collector. The cell was discharged at a current density of 100 mA g^{-1} in a potential range of 0.01~3.0 V *vs.* Li/Li$^+$.

To understand the mechanism of lithium storage in the Cu_2S/C, *operando* SRD data were collected at the MSPD beamline, ALBA, Spain using synchrotron radiation (λ = 0.41311(2) Å). LaB_6 was used as a standard sample for wavelength calibration. Coin cells with glass windows (Li||LP30||Cu_2S/C) were assembled. The working electrodes were prepared by mixing 70 wt.% Cu_2S/C. The mixture (~3.5 mg) was pressed on a copper mesh disk (ϕ 12 mm). During the *operando* SRD test, the cell was cycled at 60 mA g^{-1} in a potential range of 0.01~3.0 V *vs.* Li/Li$^+$ at room temperature.

Electrochemical characterization

The working electrodes were made of Cu_2S/C (70 wt.%), carbon black (20 wt.%) and PVDF binder (10 wt.%, R6020/1001, Solvay). The as-prepared homogeneous slurry was coated on a copper foil. The coated foils were dried in a fume hood for 2 h and then dried at 80 °C for 12 h to completely remove N-methyl pyrrolidone solvent. Afterward, the coated foils were punched into circular electrodes (ϕ 12 mm) with mass loading of 1.5~1.8 mg cm^{-2} Cu_2S/C and a drying thickness of ~12.2 µm. The assembled LIB coin cell (CR2032 type) consists of a working electrode, a Whatman® glass-fiber separator (ϕ 17 mm), a lithium foil (ϕ 15 mm, Alfa Aesar) and 180 µL of LP30 electrolyte (1 M $LiPF_6$ in ethylene carbonate/dimethyl carbonate = 1:1

by mass ratio, BASF). All cells were assembled in an argon-filled glovebox (MB200, MBraun GmbH). During the electrochemical tests, the cells were kept in a climate chamber (Binder GmbH) at 25 °C. Galvanostatic charge/discharge (GCD) and cyclic voltammetry (CV) were employed using a Bio-Logic VMP3 potentiostat in a potential range of 3.0 to 0.01 V *vs.* Li/Li^+.

Discussion Section

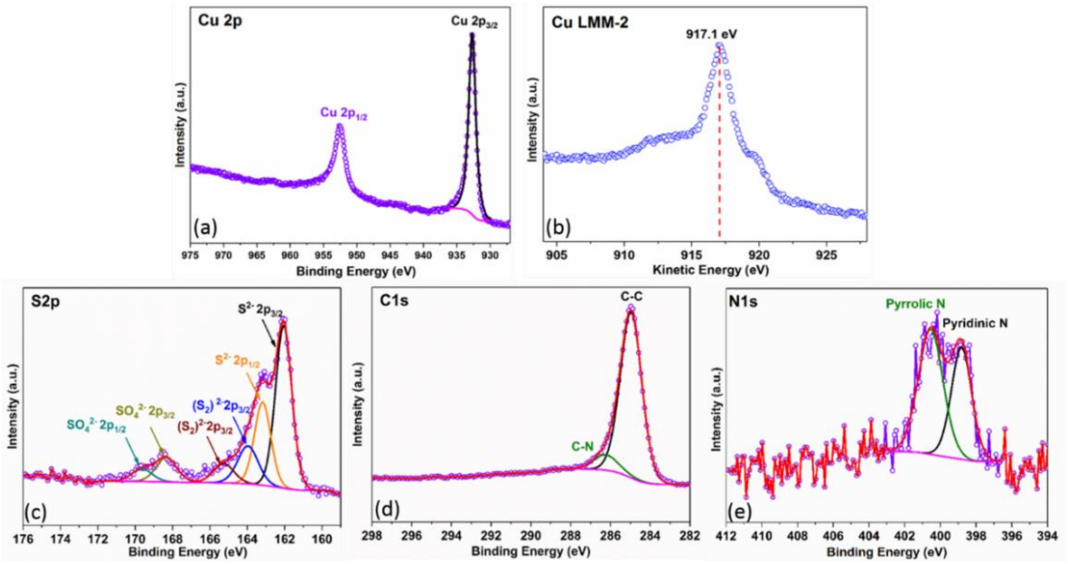

Figure S1. XPS spectra of pristine Cu_2S/C: Cu 2p (a), Cu LMM-2 (b), S 2p (c), C 1s (d), and N 1s (e).

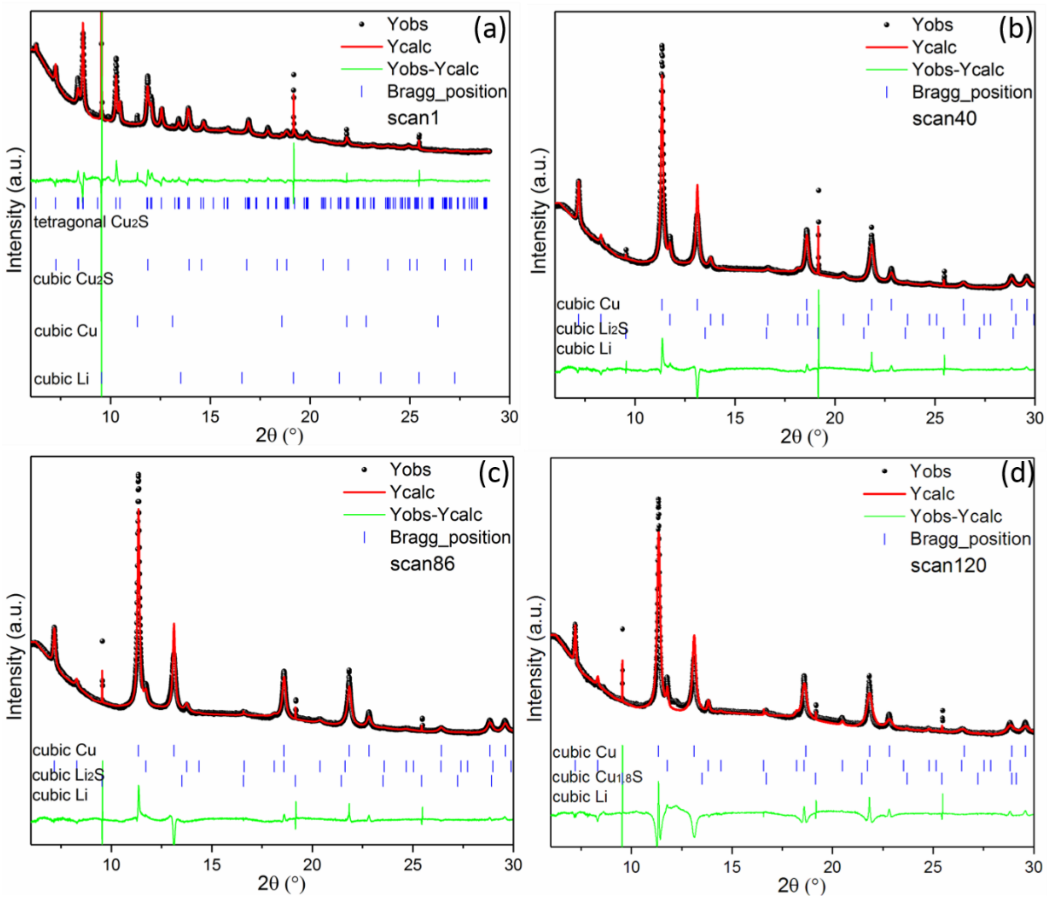

Figure S2. Analysis of typical phase change of the Cu_2S/C electrode in LIBs based on Rietveld refinement: (a) step A–scan1, (b) step B–scan40, (c) step C–scan86, and (d) step D–scan120.

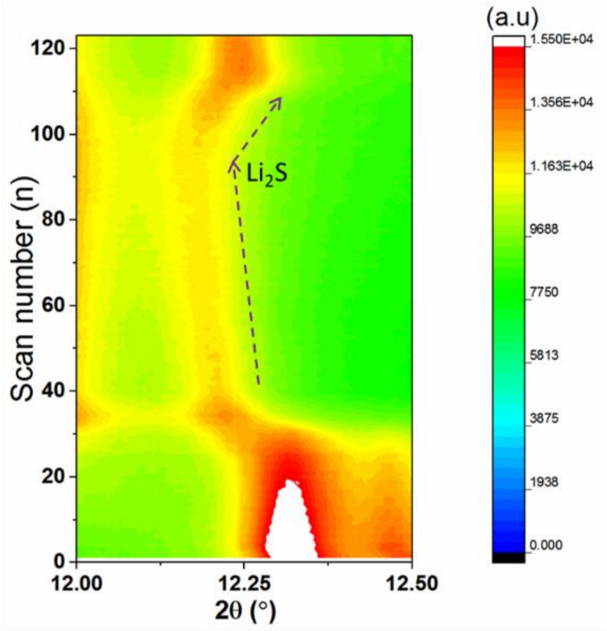

Figure S3. *Operando* SRD analysis of the Li_2S reflection in Cu_2S/C electrode.

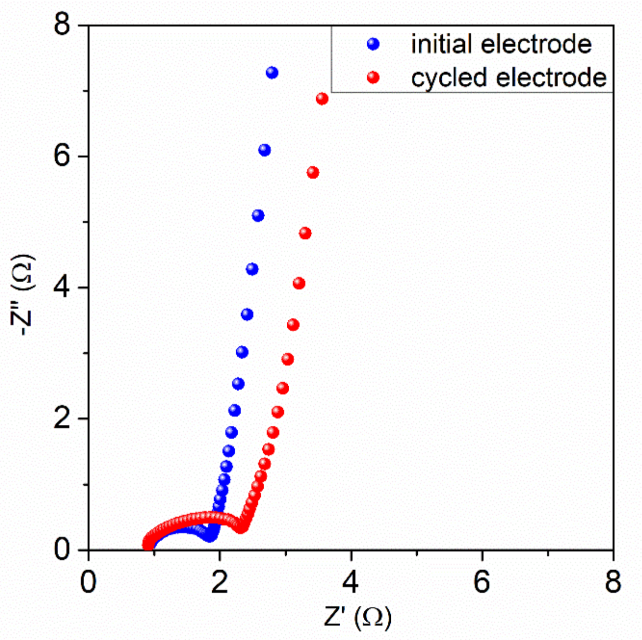

Figure S4. Nyquist plots obtained from the EIS test of the Cu_2S/C composite electrode before and after the first cycle at 60 mA g^{-1} in a potential range of 0.01~3.0 V vs. Li/Li^+ at room temperature.

Table S1. A review of preparation methods of Cu_2S and Cu_2S/carbon composite, and corresponding electrochemical performance for LIBs.

sample	Cu_2S preparation	Cu_2S morphology	compositing method	specific capacity	initial coulombic efficiency	year
Cu_2S[3]	hydrothermal approach	particles (~500 nm)	−	0.27 mAh cm^{-2} at 100 cycles at 0.1 mA cm^{-2}	99.4%	2012
Cu_2S[4]	hydrothermal method	particle cluster	−	~25 mAh g^{-1} at 0.1 A g^{-1} after 100 cycles	83%	2015
Cu_2S[5]	precipitation	spherical particles (~0.4μm)	−	~220 mAhg^{-1} at 1C over 100 cycles	~100%	2016
Cu2S[6]	hydrothermal approach	nanorods	−	164.8 mAh g^{-1} after 150 cycles at 0.2A g^{-1}	53.7%	2018
Cu_2S[7]	hydrothermal method	flower−likes	−	~300 mAh g^{-1} at 0.1 A g^{-1} after 200 cycles	~62%	2020
Cu_2S/CuS[8]	commercially available	rods (~3 mm in diameter and 6 mm in length)	mechanical stirring	~200 mAh g^{-1} at 1C for over 150 cycles	>98.4%	2014
Cu_2S/tubular mesoporous carbon[9]	in situ electrochemical corrosion	Cu_2S/tubular mesoporous carbon composite	direct growth of Cu_2S nanowire on a Cu film	270 mA h g^{-1} at 0.2C after 300 cycles	92 %	2014
Cu_2S/carbon composites[4]	hydrothermal method	particle cluster	calcination with glucose	300 mAh g^{-1} after 100 cycles at 0.1A g^{-1}	72%	2015
Cu_2S@ N, S-doped carbon[6]	hydrothermal approach	nanorods	in−situ polymerization and carbonization with pyrrole	560 mAh g^{-1} at 1A g^{-1} after 550 cycles	62.4 %	2018
Cu_2S@rGO[7]	hydrothermal method	flower−likes (thickness ~20nm)	freeze−drying process	600 mAhg^{-1} after 200 cycles at	98%	2020

				0.1 Ag^{-1}		
Cu_2S/C[10]	spray-dried	~10 nm particles	co-calcination with glucose	250 mAh g^{-1} at 0.2 A g^{-1} after 100 cycles	> 98%	2020
Cu_2S/N-doped carbon[this work]	precipitation	~90 nm particles	co-calcination with polyelectrolytes	523 mAh g^{-1} at 0.1 A g^{-1} at the 200th cycle	57.9%	2021

References

[1] G. Tian, F. Scheiba, L. Pfaffmann, A. Fiedler, V. S. K. Chakravadhanula, G. Balachandran, Z. Zhao, H. Ehrenberg, *Electrochim. Acta* **2018**, *283*, 1375–1383.

[2] J. Rodríguez-Carvajal, *Phys. B Condens. Matter* **1993**, *192*, 55–69.

[3] S. Ni, T. Li, X. Yang, *Thin Solid Films* **2012**, *520*, 6705–6708.

[4] Y. Liu, B. Jin, Y. F. Zhu, X. Z. Ma, X. Y. Lang, *Int. J. Hydrogen Energy* **2015**, *40*, 670–674.

[5] G. Kalimuldina, I. Taniguchi, *J. Power Sources* **2016**, *331*, 258–266.

[6] Q. Chen, M. Ren, H. Xu, W. Liu, J. Hei, L. Su, L. Wang, *ChemElectroChem* **2018**, *5*, 2135–2141.

[7] X. Zhang, L. Duan, X. Zhang, X. Li, W. Lü, *J. Alloys Compd.* **2020**, *816*, 152539.

[8] B. Jache, B. Mogwitz, F. Klein, P. Adelhelm, *J. Power Sources* **2014**, *247*, 703–711.

[9] F. Han, W. C. Li, D. Li, A. H. Lu, *ChemElectroChem* **2014**, *1*, 733–740.

[10] Y. Wang, X. Feng, Y. Xiong, S. Stoupin, R. Huang, M. Zhao, M. Xu, P. Zhang, J. Zhao, H. D. Abruña, *ACS Appl. Mater. Interfaces* **2020**, *12*, 17396–17405.
